# Optimization of the Emulsifying Properties of Food Protein Hydrolysates for the Production of Fish Oil-in-Water Emulsions

**DOI:** 10.3390/foods9050636

**Published:** 2020-05-15

**Authors:** Marta Padial-Domínguez, F. Javier Espejo-Carpio, Raúl Pérez-Gálvez, Antonio Guadix, Emilia M. Guadix

**Affiliations:** Department of Chemical Engineering, University of Granada, 18010 Granada, Spain; fjespejo@ugr.es (F.J.E.-C.); rperezga@ugr.es (R.P.-G.); aguadix@ugr.es (A.G.); eguadix@ugr.es (E.M.G.)

**Keywords:** emulsifying properties, statistical modelling, optimization, protein emulsifiers, physical stability, oxidative stability

## Abstract

The incorporation of lipid ingredients into food matrices presents a main drawback—their susceptibility to oxidation—which is associated with the loss of nutritional properties and the generation of undesirable flavors and odors. Oil-in-water emulsions are able to stabilize and protect lipid compounds from oxidation. Driven by consumers’ demand, the search for natural emulsifiers, such as proteins, is gaining much interest in food industries. This paper evaluates the in vitro emulsifying properties of protein hydrolysates from animal (whey protein concentrate) and vegetal origin (a soy protein isolate). By means of statistical modelling and bi-objective optimization, the experimental variables, namely, the protein source, enzyme (i.e., subtilisin, trypsin), degree of hydrolysis (2–14%) and emulsion pH (2–8), were optimized to obtain their maximal in vitro emulsifying properties. This procedure concluded that the emulsion prepared from the soy protein hydrolysate (degree of hydrolysis (DH) 6.5%, trypsin) at pH 8 presented an optimal combination of emulsifying properties (i.e., the emulsifying activity index and emulsifying stability index). For validation purposes, a fish oil-in-water emulsion was prepared under optimal conditions, evaluating its physical and oxidative stability for ten days of storage. This study confirmed that the use of soy protein hydrolysate as an emulsifier stabilized the droplet size distribution and retarded lipid oxidation within the storage period, compared to the use of a non-hydrolyzed soy protein isolate.

## 1. Introduction

Oil-in-water emulsion systems are found in numerous natural and processed foods, such as milk, salad dressings, ice cream or butter, among others [1]. Besides, emulsions are increasingly used as delivery systems to encapsulate, protect and release functional ingredients into a food matrix. Such an example is omega-3 fortified fish oils [2,3]. The main drawback of incorporating omega-3 fatty acids as ingredients is their oxidative instability, so oil-in-water emulsions and encapsulation processes are a vehicle to protect them [4,5].

Additionally, these food emulsions are thermodynamically unstable complex systems and they can separate into their watery and oily phase over time [6]. Due to this fact, it is necessary to have the presence of certain components called emulsifiers with amphiphilic properties, which can interact in the oil–water interface and reduce the surface tension [7]. Emulsifiers are surfactants that facilitate the formation of the emulsion and preserve its stability over time [8]. Besides maintaining the physical stability of the emulsion over time, a good emulsifier acts as a barrier against lipid oxidation [9]. Thus, the amount of emulsifier adsorbed on the water–oil interface and its structure are factors to consider regarding the physical stability of the emulsions, and the oxidative stability of the lipids contained in them, during their storage [10].

The food industry employs proteins, phospholipids and other surfactants from natural or synthetic origin as emulsifiers [11]. Driven by consumer demand for natural products, there is an increasing interest in searching for natural emulsifiers from protein sources [12]. Food proteins from either animal (e.g., caseins, whey proteins) or plant origin (e.g., soy proteins) are amphiphilic molecules able to be absorbed at the interface of the emulsion and to stabilize the oil droplets [13]. The functional properties of such proteins are mainly determined by the position and number of amino acids in the polypeptide chain [14,15]. To this regard, a good emulsifier must present an optimal balance between polar and non-polar groups for an adequate water solubility and surface activity [16], which in turn has an important effect on the physical and oxidative stability of the emulsions. Accordingly, a change in the pH of the environment alters the net charge of the protein [17] and its conformation at the interface [18], affecting their emulsifying capacities.

Besides pH, the enzymatic hydrolysis of proteins has proved to improve their solubility and emulsification capacity due to the changes in the globular structure [19]. The extent of the proteolysis is accompanied by a decrease of the molecular weight and increase of the surface activity of the peptides [20], which favors the stability and viscoelasticity of the interfacial films in the emulsions [21]. However, an extensive hydrolysis is detrimental for the emulsifying properties, since short-chain peptides cannot reorient and unfold like large molecules to stabilize emulsions [22]. For instance, some authors reported that the emulsifying properties of whey protein hydrolysates improve in the range of degree of hydrolysis between 3 and 10% [23,24], while further proteolysis had an adverse effect. Considering the specificity of proteolytic enzymes, the type of enzyme employed also influences the in vitro emulsifying properties of the resulting hydrolysates. To this regard, Rdsa et al. [25] investigated the effect of different commercial proteases (i.e., subtilisin, trypsin, flavourzyme) in the hydrolysis of whey proteins, reporting significant differences in the emulsifying and antioxidant activities of the hydrolysates.

Therefore, the emulsifying capacity of peptides derived from the enzymatic hydrolysis of proteins depends on the protein source and the hydrolysis conditions, such as degree of hydrolysis (DH), type of enzyme and pH of the environment [26,27]. The degree of hydrolysis is defined as the percentage of total peptide bonds that are cleaved in the course of the enzymatic reaction. Although the effect of pH and DH on emulsion stability is reviewed extensively in the literature, a limited number of studies evaluate the impact of those processing variables on the oxidative stability of fish oil-in-water emulsions. The objective of this work is to model the emulsifying properties of whey and soy protein hydrolysates according to the enzyme, the final DH of the hydrolysates as well as the pH of the emulsion preparation, and test them in real emulsions of fish oil in water.

## 2. Materials and Methods

### 2.1. Protein Substrates and Enzymes

Whey protein concentrate (WPC) and soy protein isolate (Soy) were obtained from Wheyco GmbH, (Hamburg, Germany) and Solae LLC, (St. Louis, MO, USA), respectively. Subtilisin (EC 3.4.21.62) and trypsin (EC 3.4.21.4), kindly donated by Novozymes (Bagsvaerd, Denmark), were the enzymes employed in hydrolysis. The refined fish oil Omevital 18/12 TG Gold was purchased from BASF Personal Care and Nutrition GmbH (Illertissen, Germany) with a minimum content of omega-3 fatty acids of 35% (18% of EPA and 12% of DHA).

### 2.2. Hydrolysis Procedure

Hydrolysates of whey and soy protein were produced using subtilisin (EC 3.4.21.62) and trypsin (EC 3.4.21.4) (Novozymes, Denmark) as enzymes. To evaluate the effect of degree of hydrolysis on the emulsifying properties, five hydrolysates of a different degree of hydrolysis (2%, 4%, 6%, 10% and 14%.) were produced with each enzyme. Hydrolysis was carried out in a jacketed stirred tank reactor that maintained a temperature at 50 °C during operation. When the protein solution (40 g/L) reached 50 °C, enzyme was added to the reactor at an enzyme/substrate ratio of 0.5%. The pH was maintained constant at pH 8 by adding NaOH 1M. The reaction was monitored by the pH-stat method [28], which relates in real time the consumption of base with the number of peptide bonds cleaved according to Equation (1):

(1) $$DH=\frac{B\cdot N_{b}}{\alpha \cdot m_{p}\cdot h_{TOT}}\cdot 100$$

where *B* (mL) is the amount of base consumed, *N_b_* (eq/L) is the normality of the base, *α* is the average degree of dissociation of the *α*-NH_2_ groups released during hydrolysis, *m_p_* (g) is the mass of protein in the substrate and *h_TOT_* (meq/g) is the number of equivalents of peptides bonds per gram of protein. After reaching the desired DH, the reaction was stopped by thermal deactivation of the enzyme at 90 °C for 5 min. Afterward, the samples were freeze-dried for further use.

### 2.3. Determination of the Emulsifying Properties

For each hydrolysate sample, the emulsifying activity and stability were determined at pH 2, 4, 6 and 8. Emulsifying activity is defined as the maximum amount of oil that can be emulsified by a fixed amount of the protein, while stability of the emulsion is defined as the rate of phase separation in water and oil during storage of the emulsion [29].

Hydrolysates samples were dissolved in ultrapure water and the pH was adjusted with 1 M NaOH or 1 M HCl. Then, 51 g of the aqueous solution at a protein concentration of 0.5% was mixed with 9 g of vegetable oil (i.e., sunflower oil) and then homogenized using an Ultra Turrax (IKA Werke GmbH &.Co., Staufen, Germany) at 16,000× rpm for 2 min. Two aliquots of the emulsion (50 µL) were pipetted at 0 and 10 min and diluted with 5 mL of a 0.1% sodium dodecyl sulphate (SDS) solution. The absorbance was determined with a 10-mm path length cuvette in a spectrophotometer (Cary 100 Bio, Varian, Palo Alto, CA, USA) at a wavelength 500 nm. The emulsifying activity index (EAI) and the emulsion stability index (ESI) were calculated by Equations (2) and (3), respectively:

(2) $$EAI\;\left(\frac{m^{2}}{g}\right)=\frac{2\cdot 2.303\cdot A_{0}\cdot DF}{\left(1-\theta \right)\;\cdot m_{P}\;}$$

(3) $$ESI\;\left(min\right)=\frac{A_{0}\cdot \Delta t}{A_{0}-A_{10}}$$

where *A*_0_ and *A*_10_ was the absorbance measured at initial time and after Δ*t* = 10 min, respectively. The variables *m_P_* and *θ* stand for the mass of protein (g) and the volume fraction of oil in the emulsion and DF is the dilution factor.

### 2.4. Statistical Modelling and Optimization

#### 2.4.1. Statistical Design and Regression Model

The effects of substrate (i.e., WPC or soy protein), enzyme (i.e., subtilisin or trypsin), final degree of hydrolysis and pH of the emulsion were related to the emulsifying properties (i.e., emulsion activity and emulsion stability indices) of the hydrolysates by means of a statistical regression model. To this end, the experimental factors were varied according to a full factorial design, involving both categorical and numerical variables. Both substrate (noted as S) and enzyme (E) are categorical variables, each one taking two levels (i.e., WPC or soy protein for S; subtilisin or trypsin for E). Emulsion pH and the final degree of hydrolysis (DH) are numerical continuous variables, which were tested at 4 levels (pH 2, 4, 6 and 8) and 5 levels (DH 2%, 4%, 6%, 10% and 14%), respectively. Therefore, the full factorial design comprised 2 (S) × 2 (E) × 4 (pH) × 5 (DH) = 80 experimental trials. For each experimental run, the emulsion activity index (EAI, m^2^/g) and the emulsion stability index (ESI, min) were determined as numerical outputs.

The nature of the input variables suggested employing a regression model with mixed categorical and continuous factors. Categorical variables can be incorporated into a regression model as numerical inputs by choosing an appropriate coding method. Among the different alternatives described in literature, the dummy coding method was chosen for this work [30,31]. By this approach, the levels of substrate were coded as 0 (soy protein isolate) and 1 (whey protein concentrate). As for the enzyme, it took the values 0 (trypsin) and 1 (subtilisin).

**Figure 1:**
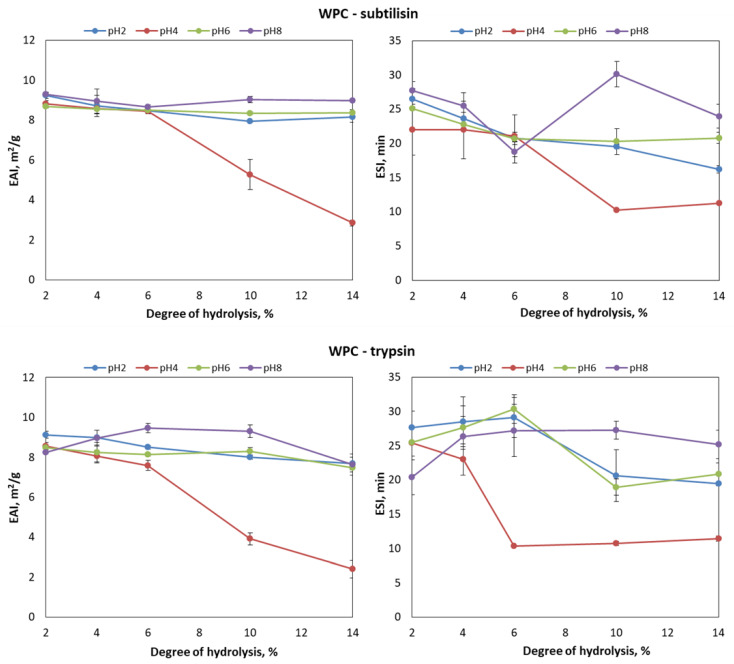
Emulsifying activity index (EAI, m^2^/g) and emulsifying stability index (ESI, min) against pH (2, 4, 6 and 8) and degree of hydrolysis (2%, 4%, 6%, 10% and 14%) for the whey protein concentrate (WPC) hydrolysates produced with subtilisin and trypsin.

**Figure 2:**
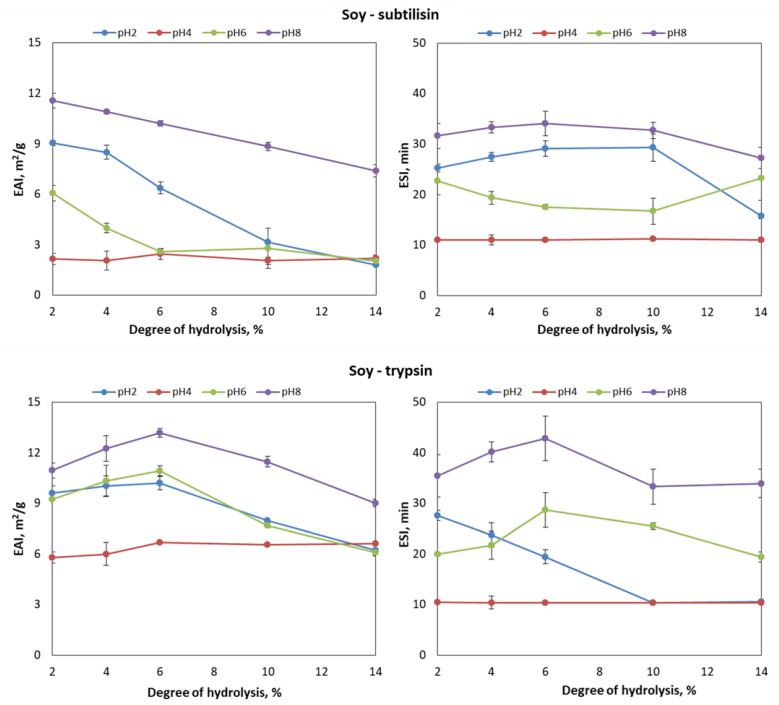
Emulsifying activity index (EAI, m^2^/g) and emulsifying stability index (ESI, min) against pH (2, 4, 6 and 8) and degree of hydrolysis (2%, 4%, 6%, 10% and 14%) for the soy hydrolysates produced with subtilisin and trypsin.

Based on the trend showed by EAI and ESI against pH and DH (Figure 1 and Figure 2), a full cubic model was proposed for the multiple regression. The full cubic model, expressed by Equation (4), comprise linear, quadratic and cubic terms for the numerical variables, as well as their binary and ternary interactions with the categorical variables.

(4) $$\begin{array}{l} \left\{EAI,ESI\right\}=b_{0}+b_{1}\cdot S+b_{2}\cdot E+b_{3}\cdot pH+b_{4}\cdot DH+b_{12}\cdot S\cdot E+b_{13}\cdot S\cdot pH+b_{14}\cdot S\cdot DH+\\ \;\;\;\;\;\;\;\;\;\;+b_{23}\cdot E\cdot pH+b_{24}\cdot E\cdot DH+b_{33}\cdot pH^{2}+b_{44}\cdot DH^{2}+b_{123}\cdot S\cdot E\cdot pH+b_{124}\cdot S\cdot E\cdot DH+\\ \;\;\;\;\;\;\;\;\;\;+b_{133}\cdot S\cdot pH^{2}+b_{134}\cdot S\cdot pH\cdot DH+b_{144}\cdot S\cdot DH^{2}+b_{233}\cdot E\cdot pH^{2}+b_{234}\cdot E\cdot pH\cdot DH+\\ \;\;\;\;\;\;\;\;\;\;+b_{244}\cdot E\cdot DH^{2}+b_{333}\cdot pH^{3}+b_{334}\cdot pH^{2}\cdot DH+b_{344}\cdot pH\cdot DH^{2}+b_{444}\cdot DH^{3} \end{array}$$

The intercept and coefficients of the full cubic model were estimated by multiple regression. An ANOVA analysis was performed to evaluate the significance of each term of the regression model. An associated probability (*p*-value) was computed for each effect (i.e., linear, quadratic, cubic or interaction term in the polynomial) at a confidence level of 95%. Those effects with *p*-values above 0.05 were not considered as statistically significant on the responses of EAI or ESI, and could be removed from the full cubic model. The backward selection method [32] was chosen for this purpose. This approach consists of sequentially removing those effects with a higher *p*-value until obtaining a final reduced model, where all the terms are significant (i.e., *p*-value < 0.05) on the response variable.

#### 2.4.2. Optimization of the Emulsifying Properties

The regression models expressed by Equation (4) are third-degree polynomials, which can be optimized to obtain a maximum for each response studied (EAI, ESI). The maximization of each response variable is desirable to ensure that the hydrolysate is able to stabilize the emulsion within the storage period. According to the experimental data, the operating conditions maximizing EAI may be detrimental for the optimization of ESI. The conflicting behavior of the experimental factors suggested combining both objectives into a single function OBJ (S, E, pH, DH) according to the weighted-sum method [33]. In our case, the optimization problem was stated as finding the combination of factors (i.e., S, E. pH, DH) within their experimental range, which maximizes the objective function:

(5) $$\begin{array}{l} maximize\;OBJ(S,E,pH,DH)=w\cdot \frac{EAI}{\lambda_{1}}+(1-w)\cdot \frac{ESI}{\lambda_{2}}\\ subjected\;\mathrm{to}:\\ \;\;\;\;\;\;\;\;\;\;\;\;\;\;\;\;\;\;\;\;\;\;\;S,E\;\in \;\left\{0,1\right\}\\ \;\;\;\;\;\;\;\;\;\;\;\;\;\;\;\;\;\;\;\;\;\;\;2\leq pH\leq 8\\ \;\;\;\;\;\;\;\;\;\;\;\;\;\;\;\;\;\;\;\;\;\;\;2\leq DH\leq 14 \end{array}$$

The coefficient *w* is the weight factor, which represents the relative importance attached to a given single objective. For instance, *w* = 1 poses the single optimization of the emulsion activity index. In our case, where no single objective is predominant, *w* = 0.5. The responses EAI and ESI were normalized within the interval (0,1) by dividing by the coefficients *λ*_1_ and *λ*_2_, which are the maximal values predicted by the cubic models for the ESI (12.7 m^2^/g) and EAI (39.0 min). The evolutionary algorithm, implemented in the Solver Tool of the MS Excel software, was chosen for the optimization.

### 2.5. Emulsion Preparation and Storage Study

The hydrolysate with the best emulsifying properties was selected for studying the stability of the emulsion. The native protein without hydrolysis was also used for producing a control emulsion. Emulsifiers (hydrolysate and native protein) were dissolved in distilled water and the pH was adjusted to the optimum identified using the models previously created. Emulsions containing 2% (*w*/*w*) protein and 5% (*w*/*w*) fish oil were produced similarly to García-Moreno et al. [5]. Initially, a pre-emulsion was prepared using Ultra Turrax (IKA Werke GmbH &.Co., Staufen, Germany) at 16,000× rpm for 2 min. The oil was added slowly to the aqueous phase during the first minute. Then, the homogenization was done in a high-pressure homogenizer (Panda Plus 2000, GEA Niro Soavi, Lübeck, Germany) at 450/75 bar, running 3 passes. The temperature during the process was controlled and kept under 26 °C. To accelerate lipid oxidation, a solution of 100 mM FeSO_4_ was added to the emulsions (4 µL per 1 g of emulsion). A solution of 0.0125 g/mL of sodium azide was also added to prevent spoilage during storage. Emulsions were stored in the dark at 20 °C for 10 days in 30 mL glass bottles. Each bottle contained approximately 10 mL of emulsion. Samples were taken at days 0, 1, 3, 6 and 10 for droplet size and oxidative stability measurements.

### 2.6. Physical Stability of the Emulsions

The droplet size distribution was measured using a Malvern Mastersizer 2000 (Malvern Instruments Ltd., Worcestershire, UK). The emulsion samples were diluted in recirculating water (3000× rpm) until it reached an obscuration of 12–15%. The refractive indices of sunflower oil (1.469) and water (1.330) were used for particle and dispersant, respectively. Measurements were done in triplicate. The Sauter diameter D(3,2) was chosen as the mean diameter of the droplet size distribution.

The zeta potentials of the emulsions were estimated in a Zetasizer Nano ZS (Malvern Instruments Ltd., Worcestershire, UK). Emulsions were diluted 1:1000 in distilled water. Measurements were done in triplicate.

### 2.7. Oxidative Stability of Emulsions

To evaluate the oxidative stability of the emulsified oil, the hydroperoxide content and anisidine index were measured during the 10-day storage (0, 1, 3, 6 and 10 days). The extractions of the oil were done with a mixture of 2-propanol/hexane (1:1, *v*/*v*). The hydroperoxide value (PV) was determined using the thiocyanate assay [34]. Briefly, the extracted oil was diluted with 2-propanol and mixed with iron-II-chloride and ammonium thiocyanate solutions. Samples were incubated for 30 min at 60 °C; then, the absorbance was measured at 485 nm. Extractions were done in duplicate and the samples were done in quadruplicate. The presence of secondary oxidation compounds was determined by the anisidine value (AV), which was determined according to the standard ISO 6885:2006. The AV method is based on the reaction of p-anisidine diluted in acetic acid with the *α* and *β* unsaturated aldehydes present in the oil. Results were expressed as 100 times the increment of absorbance, measured at a wavelength of 350 nm in a 10 mm cell of the test solution when reacted with *p*-anisidine under the test conditions specified in the International Standard.

The total oxidation value (TOTOX) is a comprehensive oxidation index calculated from the weighted sum of the peroxide value (PV) and *p*-anisidine value (AV) as follows:

(6) TOTOX=2·PV+AV

### 2.8. Statistical Analysis

The analysis of variance (ANOVA) was carried out using Statgraphics (version 5.1. Statgraphics.Net., Madrid, Spain) Mean values were compared using the Tukey’s multiple range test. Differences between means were considered significant at *p* ≤ 0.05.

## 3. Results and Discussion

### 3.1. Characterization and Emulsifying Properties

Figure 1 is a plot of the in vitro emulsifying activity index (EAI) and the stability index (ESI) against DH (2–14%) and pH (2–8) for the fish oil-in-water emulsions that were stabilized with WPC and hydrolyzed with subtilisin and trypsin. For both enzymes, the levels of EAI slightly decreased with DH, presenting an average value between 8 and 10 m^2^/g. This worsening with increasing DH was more pronounced for the emulsion at pH 4, where EAI decreased by 40%. As for the ESI values, they fluctuated along the interval of DH, presenting maximal values at DH 10% and pH 8 (subtilisin) and DH 6% and pH 6 (trypsin). These results are in line with Scherze and Muschiolik (2001) [24], who reported optimal emulsifying properties for oil-in-water emulsions stabilized with WPC hydrolysates at DH 10%. The stability of this emulsion was maintained even with hydrolysates at DH 20% and DH 27%.

Overall, the emulsions stabilized with soy hydrolysates presented maximal ESI values at DH 6%. Both the formation and stability indices worsened at DH levels above this value, regardless of the enzyme employed. These results agree with Jung, Murphy and Johnson (2005) [35], who reported maximal stability for the emulsions prepared with soy hydrolysates at DH 4% and pH 7. Similarly, Lopes-da-Silva and Monteiro (2019) [36] studied the effect of DH on the emulsifying properties of soy protein isolate, concluding that the emulsifying activity was superior at low DH levels.

The emulsifying properties of hydrolysates are mostly affected by the amphiphilicity of the peptides. It is widely reported that large peptides above 2 kDa improve the emulsifying properties of hydrolysates due to their ability to unfold at the oil/water interface [37]. Furthermore, they are more likely to have both hydrophobic and hydrophilic residues that interact with the oil droplets and the aqueous phase, respectively. This interaction increases the stability of the emulsion due to steric effects [13,38]. On the other hand, small-chain peptides delivered by enzymatic hydrolysis have a partially exposed hydrophobic core, which explains its higher diffusion rate at the oil/water interface and its ability to cover a larger area of the interface [19,38,39,40]. However, a large extent of the proteolysis yields a final hydrolysate with reduced amphiphilicity, which is detrimental for the emulsion formation and stability [13,41,42].

As for the influence of pH, Figure 1 and Figure 2 show that the lowest EAI and ESI values were observed at pH 4, close to the isoelectric point of soy and whey proteins, where their solubility is lower [29,43,44,45]. Overall, pH values away from the isoelectric point improved the stability of the emulsion. This is confirmed by Figure 1 and Figure 2, which show maximal EAI and ESI values for the emulsions at pH 8, regardless of the protein substrate and enzyme employed.

According to Figure 1, the behavior of the EAI against DH and pH was similar for WPC hydrolysates obtained with subtilisin and trypsin. In contrast, the emulsifying properties of soy protein hydrolysates produced with trypsin were significantly superior. Indeed, the maximum EAI (13.17 ± 0.27 m^2^/g) and ESI (42.83 ± 4.39 min) values of the experimental series were found for soy protein hydrolyzed with trypsin at DH 6% (Figure 2). In line with these results, Zhao and Hou [46] reported that soy protein hydrolysates (DH 1–2%) produced with trypsin exhibited a better EAI than those hydrolyzed by neutrase. Similarly, other studies revealed the superior solubility and emulsifying properties of soy protein hydrolysates produced with trypsin [38,43]. This is explained by the specificity of trypsin towards bonds involving lysine or arginine carboxylic groups, releasing hydrophilic peptides that contribute to the stabilization of the oil droplets in the emulsion [46,47]. This behavior is more marked at low levels of DH, while at a DH above 8% their contribution to interphase hydrophobicity is similar to that provided by subtilisin [48].

### 3.2. Modelling and Optimization of the Emulsifying Properties

The emulsion activity and stability indices were modeled as a function of the type of the substrate and enzyme employed, as well as the degree of hydrolysis and pH. As mentioned above, the full cubic models (Equation (4)) were reduced by the backward selection method until obtaining the reduced models, where the terms with associated *p*-value lower than 5% were removed, as expressed by Equations (7) and (8):

(7) $$\begin{array}{l} EAI=21.093-4.827\cdot S-8.503\cdot PH+2.600\cdot S\cdot pH-1.794\cdot E\cdot pH+3.810\cdot E\cdot S\\ \;\;\;\;\;\;\;\;\;\;\;\;\;\;\;\;\;\;\;\;\;\;\;+1.572\cdot pH^{2}+0.014\cdot DH^{2}-0.080\cdot pH^{3}-0.304\cdot S\cdot pH^{2}+0.184\cdot E\cdot pH^{2} \end{array}$$

(8) $$\begin{array}{l} ESI=73.186+10.276\cdot E-38.974\cdot pH+7.346\cdot S\cdot pH-1.562\cdot S\cdot DH-2.077\cdot E\cdot pH\\ \;\;\;\;\;\;\;\;\;\;\;\;\;\;\;\;\;\;\;\;\;\;+0.135\cdot pH\cdot DH-13.219\cdot E\cdot S+7.390\cdot pH^{2}-0.065\cdot DH^{2}-0.390\cdot pH^{3}\\ \;\;\;\;\;\;\;\;\;\;\;\;\;\;\;\;\;\;\;\;\;\;-1.029\cdot S\cdot pH^{2}+0.084\cdot S\cdot DH^{2}+2.403\cdot E\cdot S\cdot pH \end{array}$$

The goodness-of-fit of the reduced model was confirmed by the coefficient of determination (R^2^ = 81.4 and 83.0% for the EAI and ESI, respectively). The optimization problem stated by Equation (5) was applied for the WPC and soy protein isolate separately, in order to obtain the hydrolysis conditions (enzyme, DH) and emulsion pH for a maximal EAI or ESI. All these optimal solutions are summarized in Table 1.

Since both single objectives (i.e., maximization of EAI and ESI) cannot be pursued simultaneously, they were combined into an objective function as expressed by Equation (5). The experimental factors maximizing the bi-objective function are also included in the Table 1.

Soy hydrolysates exhibited better emulsifying properties than the WPC hydrolysates. As for the enzymatic treatment, the emulsifying properties of the trypsin hydrolysates were generally superior to those obtained employing subtilisin as catalyst. As an exception, the maximal EAI of WPC (10.4 m^2^/g) was predicted for the subtilisin hydrolysate at DH 2%. Both emulsifying indices were maximal at DH 2%. The improvement of the emulsifying properties at a low DH is widely reported in the literature [13,37,38]. In contrast, the predicted emulsion stability index for soy hydrolysates (39 min) was maximal at DH 8.5%.

Overall, we can conclude that a pH of 8 led to improved activity and stability indices, except for the emulsion prepared from WPC hydrolyzed with trypsin. In this case, the emulsion presented optimal in vitro stability under acidic conditions (pH 2).

According to the optimization procedure, the emulsion at pH 8 prepared from soy hydrolysate with trypsin and DH 6.5% presented an optimal combination of process conditions to obtain the best EAI and ESI indices. The emulsifying indices predicted for this emulsion were 12.2 m^2^/g and 38.3 min. These conditions were validated experimentally by a storage study.

**Figure 3:**
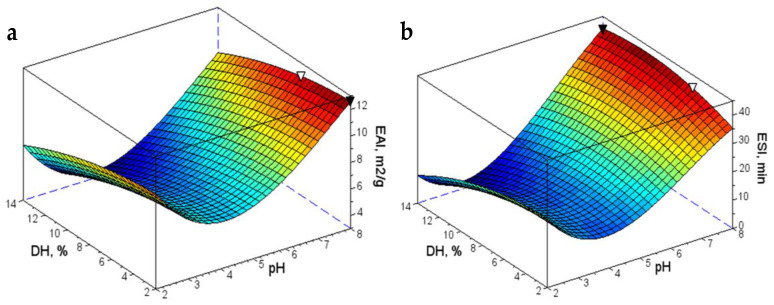
Surface plots of (**a**) EAI and (**b**) ESI for the soy hydrolysates.

The regression models expressed by Equations (6) and (7) allowed the generation of the surface plots for EAI and ESI. Figure 3 plots both properties for the soy trypsin hydrolysates as a function of pH and the degree of hydrolysis. The absolute maximums for the EAI and ESI are represented on the surface as a black marker (▼), while the white markers (▽) point out the intermediate solution optimizing both emulsifying properties.

As observed, both in vitro emulsifying properties were influenced similarly by the pH of the aqueous phase, attaining maximal levels at pH 8. Under neutral and alkaline conditions, increasing DH negatively affected the emulsion activity, while it significantly improved the emulsion stability. For both properties, a minimum was detected within the pH range 4–5, regardless the degree of hydrolysis, corresponding to the isoelectric pH for soy protein [45,46].

### 3.3. Study of Physical and Oxidative Stability of the Emulsion

#### 3.3.1. Physical Stability of the Emulsion under Optimal Conditions

The optimization procedure described above predicts an optimal emulsion at pH 8, stabilized with hydrolysates of the soy protein isolate (DH 6.5%) hydrolyzed with trypsin. For validation purposes, an emulsion was prepared under optimal conditions (coded as SPH6.5) and subjected to a storage study, where the physical and oxidative stability were monitored over ten days at 20 °C. A control emulsion (SPI), prepared with non-hydrolyzed soy protein isolate, was subjected to the same storage study.

Both optimal and control emulsions presented high initial values of zeta potential, −52.57 ± 3.26 mV and −48.71 ± 2.40 mV, respectively. The negative potential is due to the pH of the emulsions, above the isoelectric point of the protein. The results are in line with other studies in emulsions with soy protein and its hydrolysates [49,50]. A higher absolute value of the zeta potential implies a greater stability of the emulsion [51] since it reflects a better repulsion between the oil droplets, and their aggregation is also minimized [52]. In general, with zeta potential values higher than 30 mV or less than −30 mV, aggregation particles is prevented due to their electrostatic repulsions [53].

**Figure 4:**
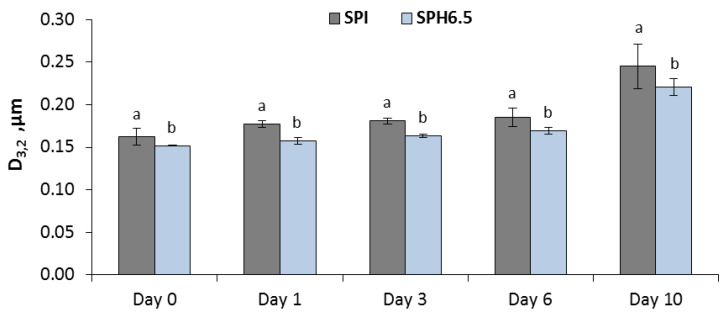
Droplet size of emulsions stabilized with a soy protein isolate (SPI) and soy protein hydrolysate (SPHT6.5) during storage at 20 °C. * Different letters represent significant differences between SPI and SPH6.5 (*p* < 0.05).

Figure 4 shows the evolution of the droplet size distribution, represented by D_3,2_ throughout the storage period. It is observed that the droplet size distribution of both emulsions was stable until the sixth day of storage. Indeed, the reference diameter D_3,2_ increased continuously over time at a rate of 0.02 μm per day until Day 6. From this day on, droplet size underwent a slight increase of 0.07 μm up to 0.221 ± 0.01 μm (Figure 4). The control emulsion (SPI) presented a similar behavior during the storage period, showing a slight tendency towards a bimodal distribution on Day 10, where a second peak centered at D(3,2) = 7.4 μm was detected (data not shown). The coalescence of oil droplets into larger units shows physical destabilization of the emulsion system, which could be accompanied by increased lipid oxidation. As shown in Figure 4, the Sauter diameters for the optimal emulsion were significantly lower than those of the control emulsion throughout the storage period. We concluded that the hydrolysis of the soy protein isolate improved its emulsifying properties with respect to the native protein.

Similar results were obtained for an oil-in-water emulsion stabilized with rice protein hydrolyzed with trypsin (DH 6.36%) [54]. In this study, there was an increase of 0.04 μm in the droplet size during storage. On Day 1 of storage the D_3,2_ was 0.27 ± 0.01 μm, and 0.31 ± 0.02 μm on Day 7 [54]. To achieve good physical stability, it is important that the fortified emulsions (i.e., emulsions enriched with Omega-3 fatty acids) have an average droplet size below 0.5 μm [3,55]. Previous studies with non-hydrolyzed vegetable protein report average droplet diameters from 0.3 μm [55,56,57,58] up to more than 1 µm [49,50].

#### 3.3.2. Oxidative Stability of the Emulsion under Optimal Conditions

**Figure 5:**
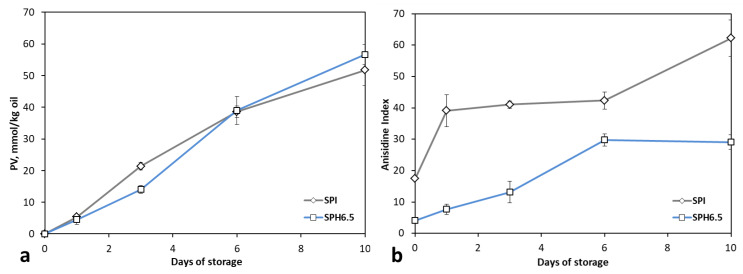
Evolution of (**a**) peroxide value (mmol/kg oil) and (**b**) anisidine index for the optimal (SPH6.5) and control (SPI) emulsions.

The peroxide value of the oil extracted from the emulsions SPI and SPH6.5 was monitored throughout the storage study (Figure 5a) in order to evaluate its oxidative stability. For the optimal SPH6.5 emulsion, the initial peroxide value (PV) is similar to that obtained for refined fish oil (<1 mmol O_2_/kg oil). It is widely accepted that the emulsification process increases the susceptibility of oil droplets to lipid oxidation [6] since it increases the contact between the polyunsaturated fatty acids with oxygen and other pro-oxidative agents. In this regard, the interface acts as an effective barrier to the oxidative stability of the emulsion [59]. In our case, this effect could be enhanced by the emulsifying and antioxidant properties of the soy protein hydrolysate, which has been reported in the literature [60,61,62,63,64,65,66].

As shown in Figure 5a, we observed that the PV for the SPH6.5 emulsion showed a constant increase from the first day of storage, until reaching a value of 56.65 mmol/kg oil on Day 10. A similar trend was also found for the evolution of the concentration of primary oxidation compounds in rapeseed oil emulsion stabilized with fava bean hydrolysate [67], in a fish oil-in-water emulsion stabilized with soy *β*-conglycinin hydrolysate [68] and with lecithin, widely used as a natural emulsifier. In stabilized oil-in-water emulsions with lecithin, the peroxide concentration increased during longer storage [69]. The authors indicate that it is possible that endogenous Fe^2+^ or Fe^3+^ ions bound to the lecithin layer in the emulsion through electrostatic attraction between the negatively charged lecithin-coated droplets and the positively charged iron ions could promote the lipid oxidation of the emulsions [69]. Differences in the PV values between our work and the latter studies are due to the incubation process carried out in our analyses to determine PV.

The PV levels of the control emulsion (SPI) increased at a higher rate than SPH6.5 up to the third day of storage. From this point on, the values of the PV were slightly lower than those observed for SPH6.5. Finally, we observed no significant differences between emulsions at the end of the storage study. Although this trend could reflect higher stability of the SPI against lipid oxidation, it does not agree with the evolution shown by anisidine index (Figure 5b). Indeed, the SPI presented higher levels of anisidine values throughout the storage period and underwent a sharp increase above the sixth day. The conflicting behavior between the peroxide and anisidine values shown by the SPI sample can be explained by the progress of oxidation reactions. Lipid oxidation results in primary peroxide compounds, which are further decomposed into secondary products. The latter cannot be quantified by the PV analysis, but are reflected in the anisidine index. García-Moreno et al. [5] also reported this trend, concluding that the rate of decomposition of peroxides into secondary products was higher than the formation rate.

**Figure 6:**
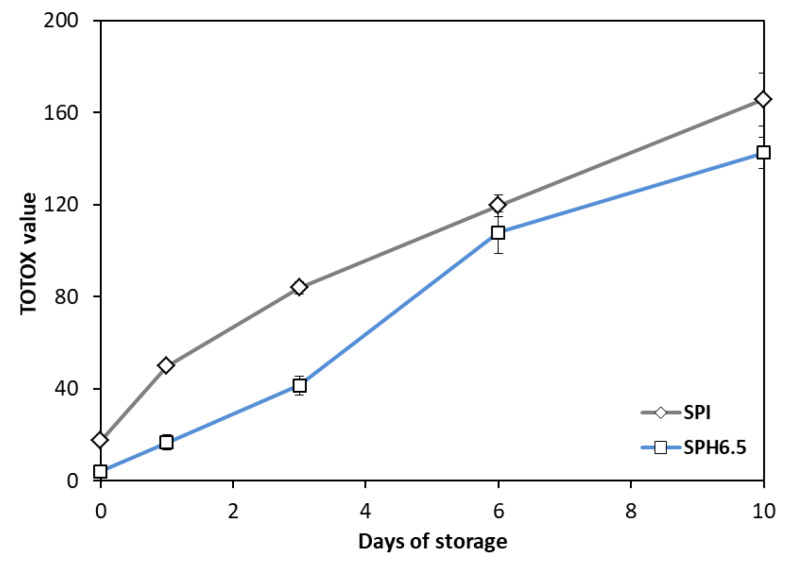
Evolution of Totox value for the optimal (SPH6.5) and control (SPI) emulsions.

The higher oxidative stability shown by the soy protein hydrolysate (SPH6.5) is confirmed by the evolution of the Totox value (Figure 6), which quantifies the global generation of oxidation products during processing. According to this figure, the SPH6.5 sample exhibited higher stability against lipid oxidation throughout the storage period. The sharp increase between Days 3 and 6 is also reflected in this index, although the overall Totox values of the hydrolysate were inferior to those observed for the protein isolate (SPI).

The anisidine value (AV) measures the secondary oxidation compounds and it is more sensitive to aldehydes, principally 2-alkenals and 2, 4-alkadienals, present in the emulsified oil [70,71]. Emulsion control showed an AV significantly higher than optimal emulsion (Figure 5b). That could be due to the fact that hydrolysis partially releases hydrophobic groups of the protein and the hydrolysate could show efficient free radical scavenging capacity and iron chelating activity compared to the intact protein [63,72,73]. Peptides derived from hydrolysis, due to their structure, are less prone to conformational folding than proteins and this ensures that most of their reactive groups are always available for antioxidant reactions [40].

## 4. Conclusions

Protein hydrolysate with improved emulsifying properties were produced from different protein sources (whey and soy protein) using subtilisin and trypsin as enzymes. The effect of substrate, enzyme, degree of hydrolysis and the pH on the emulsifying properties of hydrolysates was assayed. Generally, soy hydrolysates seemed to present higher emulsifying properties than whey protein hydrolysates. However, because of the complexity of the data, a mathematical model including all the variables was proposed for optimizing the emulsifying activity index (EAI) and the emulsion stability index (ESI). A maximum EAI of 12.7 m^2^/g was obtained for the soy hydrolysate at DH 2% produced by trypsin, while the maximum ESI (39 min) was identified for the soy hydrolysate at DH 8.5%. In order to undertake the bi-objective optimization problem, which involves both having conflicting results, the weighted-summed method generated an optimal solution, taking into account that both objectives have the same importance. According to this, the emulsion at pH 8 prepared from the soy hydrolysate with trypsin and a DH of 6.5% presented an optimal combination of emulsifying properties. The emulsion prepared under the optimal conditions proposed presented steady values of the Sauter diameter within the first six days of storage, always below those corresponding to the emulsion employing the soy protein isolate. Moreover, the hydrolysate was able to retard lipid oxidation from Day 0 to 6 of storage when compared to the use of the non-hydrolyzed soy protein isolate. In this line, these emulsions could be incorporated into certain food matrices, such as mayonnaise or salad dressings, to enrich them with omega-3 fatty acids.

## Figures and Tables

**Table 1:** The optimal substrate, enzyme and hydrolysis parameters (pH, DH) for the maximal EAI, ESI or combined objective.

Objective	* S	* E	pH	* DH, %	*EAI, m^2^/g	* ESI, min
Optimal EAI	WPC	Subtilisin	8	2	10.4	26.3
	Soy	Trypsin	8	2	12.7	36.5
Optimal ESI	WPC	Trypsin	2	2	8.8	29.7
	Soy	Trypsin	8	8.5	11.8	39.0
Optimal combined properties (EAI + ESI)	Soy	Trypsin	8	6.5	12.2	38.3

* S: Substrate; E: Enzyme; DH: Degree of Hydrolysis; EAI: Emulsifying Activity Index; ESI: Emulsifying Stability Index.
